# Post-Traumatic Stress and Stressor-Related Disorders in Hematological Malignancies: A Review

**DOI:** 10.3390/jcm14176132

**Published:** 2025-08-29

**Authors:** Adela Georgiana Buciuc, Zelde Espinel, Mary Weber, Sabrina Tran, Maria Rueda-Lara

**Affiliations:** 1 Jackson Memorial Hospital, University of Miami, 1611 NW 12th Ave., Miami, FL 33136, USA; 2 Miller School of Medicine, University of Miami, 1600 NW 10th Ave., Miami, FL 33136, USA; z.espinel@miami.edu (Z.E.); mxw808@miami.edu (M.W.); sht36@med.miami.edu (S.T.); mrueda2@med.miami.edu (M.R.-L.)

**Keywords:** PTSD, review, psychological distress, hematological cancer, mental health, trauma-related disorders, and quality of life

## Abstract

**Background:** Patients with hematological malignancies undergo intensive treatments, endure prolonged hospitalizations, and face the stress of a life-threatening diagnosis, placing them at high risk for developing post-traumatic stress disorder (PTSD) and related trauma symptoms. **Methods:** This narrative review synthesizes findings from PubMed-indexed studies examining the prevalence, clinical features, and consequences of PTSD in patients with hematological malignancies. A separate focused search was also conducted to identify PTSD studies in patients undergoing hematopoietic stem cell transplantation, which is a population recognized as being at high psychological risk. **Results:** Evidence indicates that a substantial proportion of these patients develop full or subthreshold PTSD. Key contributing factors include treatment intensity, fear of relapse, and extended hospital stays. PTSD symptoms are linked to reduced treatment adherence, diminished quality of life, and poorer clinical outcomes. **Conclusions:** Psychiatric care plays a critical role in addressing PTSD in this population. Routine trauma-informed screening, access to evidence-based pharmacologic and psychotherapeutic interventions, and close interdisciplinary collaboration with hematology teams are essential to improving patient outcomes.

## 1. Introduction

**Figure 1:**
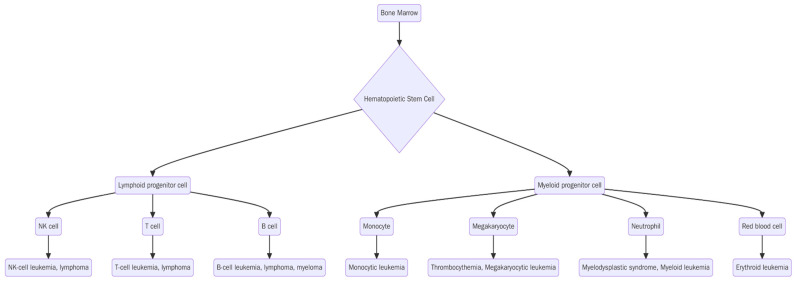
Overview of hematologic malignancies.

Hematological malignancies encompass a biologically diverse group of clonal hematopoietic disorders, broadly classified into myeloid and lymphoid neoplasms based on the lineage of origin [1]. These include acute leukemias, lymphomas, myelodysplastic syndromes (MDS), myeloproliferative neoplasms (MPNs), and plasma cell dyscrasias. Figure 1 provides an overview of the major categories of hematological malignancies based on their cellular origin. While advances have been made in the diagnosis and treatment of these cancers, aggressive treatment, unpredictable disease courses, and severe side effects have all contributed to psychological distress in patients [2]. The psychological and social burden of a cancer diagnosis has led to many patients experiencing higher levels of anxiety and depression than the general population, which can then adversely affect treatment, recovery, and outcomes. The efficacy of psychosocial interventions to treat patients with hematologic cancers who are simultaneously experiencing depression and anxiety has been established in the literature [2,3,4].

Post-Traumatic Stress Disorder (PTSD) remains an important yet underrecognized concern in this population. PTSD is a prevalent trauma- and stressor-related disorder, defined by six core diagnostic criteria as outlined in the Diagnostic and Statistical Manual of Mental Disorders, Fifth Edition, Text Revision (DSM-5-TR): (1) exposure to actual or threatened death, serious injury, or sexual violence; (2) intrusive symptoms such as distressing memories, nightmares, or flashbacks; (3) persistent avoidance of stimuli associated with the traumatic event, including places, people, and activities; (4) marked alterations in arousal and reactivity, including irritability, hypervigilance, sleep disturbance, or concentration difficulties; (5) negative alterations in cognitions and mood, encompassing distorted blame, persistent negative beliefs, emotional numbing, depressive symptoms and a duration of symptoms lasting more than one month, causing significant distress or functional impairment in social, occupational, or other important areas of life [5].

Although earlier PTSD diagnostic frameworks excluded medical conditions and treatments as qualifying traumatic events, recent updates recognize that life-threatening illnesses such as cancer and their treatments can constitute trauma capable of triggering PTSD. However, evidence on cancer-related PTSD remains limited. The application of the PTSD model in oncology has been debated due to conceptual and methodological concerns [6]. A comprehensive psychological assessment must consider pre-existing trauma or psychiatric conditions and differentiate PTSD from other diagnoses, such as adjustment disorders. Effective management of trauma-related symptoms in cancer care requires careful differential diagnosis and individualized therapeutic planning.

A diagnosis of a hematologic malignancy is often an abrupt and deeply distressing event, setting off a series of medical procedures that are physically intense, emotionally taxing, and potentially traumatic. Treatments such as high-dose chemotherapy, radiation, bone marrow or stem cell transplantation demand significant physical endurance and frequently involve prolonged hospitalization, strict isolation, and distressing medical complications. An unexpected life-threatening cancer diagnosis can disrupt a person’s sense of safety and control, forcing them to confront their mortality while undergoing invasive treatments under significant emotional strain. The psychological toll of this experience, marked by fear, uncertainty, isolation, and loss of normalcy can overwhelm coping mechanisms and place patients at risk for PTSD. Cancer-related PTSD is often characterized by a persistent fear of recurrence, with routine medical settings acting as potent trauma reminders. Patients may report intrusive thoughts or nightmares about their diagnosis or treatment, avoid follow-up care, and experience persistent hypervigilance concerning bodily symptoms and test results. Associated symptoms include depressive symptoms, distorted cognitions about the illness, and difficulties in expressing emotional distress, particularly in clinical interactions [7].

Beyond the psychological burden, patients with hematologic malignancies face a wide range of side effects that vary with disease type, treatment regimen, and individual patient factors. Acute toxicities are common, including myelosuppression, which increases the risk of infection, fatigue, and bleeding as well as gastrointestinal complications such as nausea, diarrhea, and mucositis [8,9,10]. Long-term and cumulative toxicities such as organ dysfunction, peripheral neuropathy, and cognitive impairment can significantly impact quality of life and functional recovery [11,12]. In addition, multiple disease relapses are frequent, with each recurrence can reactivate prior traumatic experiences and contribute to a sustained state of psychological distress, marked by heightened fear, hypervigilance, and ongoing concern for their health and survival.

Despite the high levels of distress reported by many patients, PTSD symptoms in this group are frequently underdiagnosed or misinterpreted as general adjustment difficulties related to cancer. Even in the absence of full PTSD, subthreshold symptoms can significantly impair quality of life, emotional well-being, and treatment adherence [13]. It is also important to recognize that many patients may exhibit substantial trauma-related symptoms without meeting full criteria for PTSD. Subthreshold or partial PTSD presentations are common and may still significantly impact functioning. Similarly, Acute Stress Disorder (ASD) may be observed in the immediate aftermath of a cancer diagnosis, particularly during the highly stressful diagnostic and initial treatment phases. According to the DSM-5-TR, ASD is characterized by the presence of nine or more symptoms from five categories: intrusion, negative mood, dissociation, avoidance, and arousal—that emerge within three days to one month following exposure to a traumatic event [5]. These symptoms must cause clinically significant distress or impairment and be directly attributable to the stressor. In oncology populations, patients may present with dissociative episodes, hyperarousal, intrusive cancer-related thoughts, or intense emotional numbing shortly after diagnosis. Although these symptoms may initially resemble those of PTSD, they often resolve spontaneously or evolve into another diagnostic entity if they persist beyond one month.

Adjustment Disorder (AjD) is another critical diagnostic consideration in cancer care. It is defined by the development of emotional or behavioral symptoms in response to an identifiable stressor such as a cancer diagnosis or treatment occurring within three months of the onset of that stressor [5]. The symptoms, which may include anxiety, depressed mood, or behavioral disturbances, must be out of proportion to the severity or intensity of the stressor and cause significant impairment in social, occupational, or other important areas of functioning. Unlike PTSD or ASD, AjD does not require exposure to a life-threatening or traumatic event per se, making it particularly relevant in the oncology context where psychological distress can arise from non-traumatic yet deeply disruptive experiences such as prognostic uncertainty, physical decline, or existential concerns that may greatly influence a patient’s overall well-being and their resilience within their cancer journey. However, diagnosing AjD can be challenging, especially when patients display both impairment (e.g., in work or social life) and resilience (e.g., strengthened family relationships or personal growth) [13].

Understanding and addressing PTSD in individuals with hematologic malignancies has profound clinical and public health implications. By identifying trauma-related symptoms early and integrating trauma-informed care into oncology and survivorship services, clinicians can help improve patient outcomes, enhance quality of life, and reduce the long-term psychological burden of cancer [13]. In turn, this could lead to more consistent treatment adherence, reduced healthcare costs, and better reintegration into family, work, and community life.

This review addresses a critical knowledge gap. While psychological distress is widely reported in hematologic oncology, the prevalence, clinical features, and the treatment implications of PTSD and related disorders remain underrecognized and not well synthesized. The primary objective of this review is to examine the prevalence and contributing factors of PTSD in adults with hematologic malignancies, including those undergoing hematopoietic stem cell transplantation (HSCT). Secondary objectives include highlighting current screening practices, diagnostic considerations, and psychosocial and pharmacologic interventions relevant to this population. By consolidating existing literature, we aim to inform trauma-informed clinical practice and identify future research priorities.

## 2. Methods

This narrative review synthesizes current research on PTSD and trauma-related symptoms in adult patients with hematologic malignancies. A narrative review methodology was chosen due to the heterogeneity of study designs, sample characteristics, and outcome measures in this field. Given the relatively limited volume of literature, this approach allows an integrative discussion of findings across diverse contexts.

Two separate literature searches were conducted in January 2025 using PubMed, Google Scholar, PsycINFO, Embase and Scopus databases. The first aimed to identify studies examining PTSD and trauma-related disorders in adults with hematologic malignancies. Keywords included “hematologic malignancies” and “post-traumatic stress disorder” or “trauma-related disorders”. The second focused specifically on PTSD in the context of HSCT, a subpopulation known to face unique psychological challenges and it was conducted in January 2025 as well. Keywords included combinations of “hematopoietic stem cell transplantation” or “HSCT” with “post-traumatic stress disorder” or “trauma-related disorders”.

We searched publications from 1 January 2015 to reflect the contemporary oncology landscape (implementation of immunotherapies, targeted therapies, survivorship programs) and maintain applicability to current clinical practice. Inclusion criteria were studies published in English, reporting original empirical data, and involving adult populations with hematologic malignancies or post-HSCT. Only quantitative studies, such as observational, prospective, retrospective, or secondary analyses were included. Studies were excluded if they were qualitative, single case reports, reviews, editorials, conducted in pediatric populations, or not published in peer-reviewed sources. The gray literature, such as conference abstracts, preprints, and dissertations were also excluded. Title and abstract screening were conducted independently by two reviewers, with full-text screening and final inclusion decisions made collaboratively.

## 3. Results

**Figure 2:**
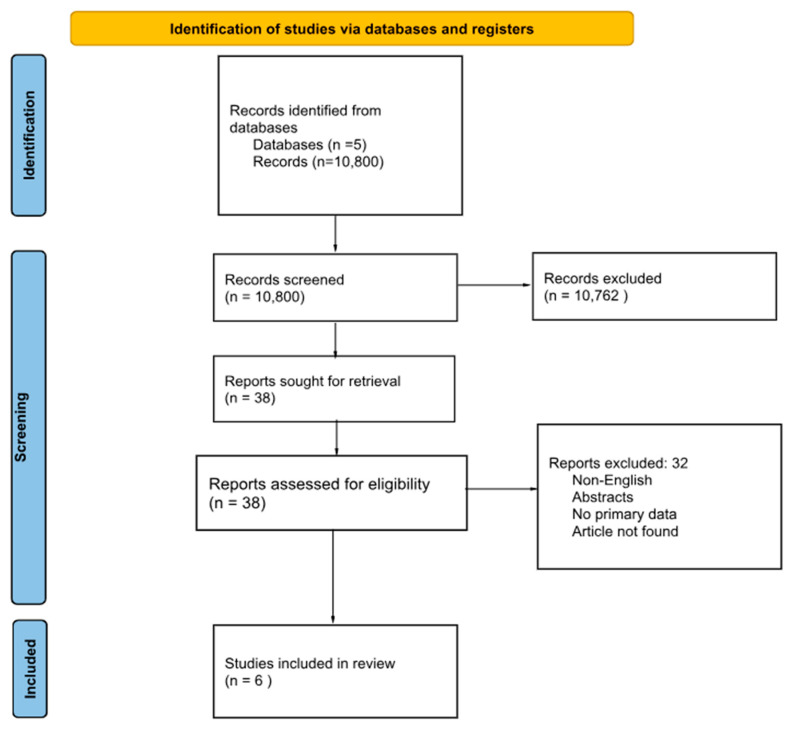
PRISMA flow diagram for PTSD and trauma-related disorders in hematologic malignancies [20].

The initial literature search identified 10,800 articles. After screening titles and abstracts, 10,762 records were excluded, and 38 articles were selected for full-text review. Thirty-two of these were excluded for the following reasons: non-English language, abstract only, no primary data, or article not found. The remaining six studies met the inclusion criteria and were included in the review. These are summarized in Table 1, and the selection process is illustrated in Figure 2 (PRISMA flow diagram).

**Figure 3:**
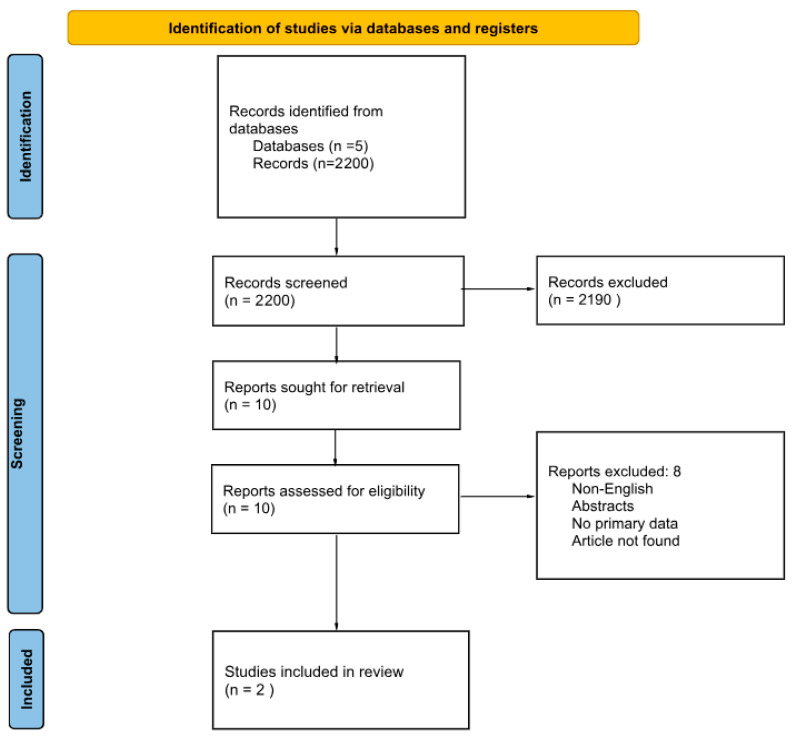
PRISMA flow diagram for PTSD after HSCT literature search [20].

The targeted search focusing on hematopoietic HSCT and PTSD identified 2200 records. After title and abstract screening, 2190 records were excluded. Ten full-text reports were assessed for eligibility, and 8 were excluded for the same reasons as above. Two studies were included and are summarized in Table 2, with the selection process being shown in Figure 3.

We conducted a qualitative appraisal of methodological rigor across five domains (design, sampling/setting, PTSD measurement, confounding control, and timing/follow-up) and synthesized findings narratively, focusing on factors likely to influence prevalence estimates and treatment effects. No formal risk-of-bias assessment was performed.

PTSD rates in hematology cohorts varied nearly fivefold, ranging from 8% in a multicenter ICU population where diaries were routinely used [17] to 36% among outpatients receiving chemotherapy during the COVID-19 pandemic [18]. Full-threshold figures were consistently accompanied by sub-threshold PTSD, which doubled or tripled caseloads—for example, 23.7% sub-threshold versus 9.3% full PTSD [15]

Methodological rigor was mixed: studies relied on self-report screeners with variable cutoffs and alignment to DSM criteria, limiting comparability of prevalence estimates. Study designs were predominantly observational and cross-sectional, using convenience samples with sparse reporting of nonresponse or attrition. Adjustment for key confounders—such as prior trauma, mood comorbidity, treatment intensity, and socioeconomic factors—was inconsistent. Contextual factors, including early post-diagnosis timing, ICU diary use, and the COVID-19 pandemic, further contributed to heterogeneity.

## 4. Discussion

### 4.1. Assessment and Screening

Across included studies, assessments relied exclusively on self-report screening instruments rather than clinician-administered diagnostic interviews. The gold standard for PTSD diagnosis remains the Clinician-Administered PTSD Scale (CAPS) or the Structured Clinical Interview for DSM-5 (SCID-5), which ensure standardized diagnosis aligned with DSM-5 criteria [23,24]; however, none of the reviewed studies used these methods. This limits diagnostic certainty and reduces comparability across prevalence estimates.

A range of validated self-administered questionnaires was employed, each varying in scope, diagnostic alignment, and clinical utility:

- Post-Traumatic Stress Disorder Checklist–Civilian Version (PCL-C)—a self-administered questionnaire widely used in cancer-related PTSD screening due to its brevity, alignment with DSM-IV criteria, and sensitivity to changes in symptom severity over time [25]. It can be used for both provisional diagnosis and monitoring of symptom progression in clinical or research settings.
- Impact of Event Scale–Revised (IES-R)—a self-administered measure capturing subjective distress in response to a specific traumatic event, assessing intrusion, avoidance, and hyperarousal [26]. While useful for screening, it does not assess all DSM-5 PTSD criteria and should be supplemented with structured diagnostic interview.
- Stanford Acute Stress Reaction Questionnaire (SASRQ)—a self-administered tool measuring acute stress symptoms in the immediate post-diagnosis phase, including dissociation, re-experiencing, avoidance, and arousal [27]. It is particularly relevant during the early post-diagnosis period, when symptoms may not yet meet the threshold or duration required for a PTSD diagnosis.
- Impact of Event Scale–Revised (IES-R)—a self-administered measure capturing subjective distress in response to a specific traumatic event, assessing intrusion, avoidance, and hyperarousal [26]. While useful for screening, it does not assess all DSM-5 PTSD criteria and should be supplemented with structured diagnostic interviews.
- Adjustment Disorder New Module 20 (ADNM-20)—a self-administered questionnaire designed to assess adjustment disorder symptoms and severity across multiple stress-related domains [28]. It is well-suited to oncology settings for identifying patients experiencing significant distress who do not meet criteria for PTSD or acute stress disorder.
- Posttraumatic Diagnostic Scale (PDS)—a self-administered questionnaire assessing DSM-IV PTSD criteria, including trauma exposure, symptom severity, and functional impairment [25]. Originally developed for non-medical trauma populations, it has demonstrated utility in oncology settings for identifying both full and subthreshold PTSD.
- PTSD Checklist–Specific (PCL-S)—a self-administered questionnaire measuring PTSD symptoms related to a specific traumatic event, used in French-validated form in Study 6 [19].

In the reviewed studies, the PCL-C was the most frequently applied tool [15,16,19,21,22]. In Study 6, it identified subthreshold PTSD in 23% of participants despite no cases meeting full diagnostic criteria, underscoring its utility for symptom monitoring [19]. In Study 3, the PCL-C was administered one-month post-diagnosis, with 25% screening positive—an interval that may capture acute stress reactions rather than persistent PTSD [16].

The IES-R was used in Studies 4 and 5 [17,18]. Study 4 applied a threshold > 35, with 8% screening positive among ICU survivors with hematologic malignancies; concurrent ICU-diary practices may have attenuated symptoms [17]. Study 5 reported a 36% positive rate, likely influenced by pandemic-related stressors [18].

The ADNM-20 was used in Study 2 to identify a clinically relevant subgroup meeting criteria for adjustment disorder but not PTSD [15]. This tool is useful for differential diagnosis and stepped-care planning in oncology settings [14].

When integrated into routine oncology care, screening tools can support early identification, differential diagnosis, and targeted intervention for trauma-related psychopathology throughout the cancer continuum [14,29].

### 4.2. Prevalence of PTSD in Hematological Malignancies

The six studies included in this review show important trends in PTSD symptomatology. Liu et al. reported a 10.7% prevalence of PTSD symptoms among Chinese patients with hematological malignancies, based on assessments using the PCL-C [17,18]. The study also examined psychological resilience factors such as hope and optimism, measured with the Chinese versions of the Adult Hope Scale (AHS) and the Life Orientation Scale–Revised (LOT-R). Optimism emerged as a protective personality trait, showing a significant negative correlation with PTSD symptom severity. These findings emphasize the potential role of psychosocial buffers in mitigating trauma-related distress in oncology settings.

Springer et al. conducted a cross-sectional study of 285 patients with hematological malignancies receiving various treatment regimens [15]. PTSD symptoms were assessed using the PCL-5, which evaluates current symptomatology in alignment with DSM-5 criteria and allows identification of subthreshold PTSD when at least two symptom clusters are present. The study found that 9.3% of participants met criteria for PTSD, 23.7% had subthreshold PTSD, and 14.2% met criteria for adjustment disorder [15]. This stratified approach revealed that over 40% of the cohort experienced clinically significant distress. Younger age, the presence of physical comorbidities, and active disease were significantly associated with higher symptom burden [15].

Amonoo et al. conducted a secondary analysis within a supportive care trial involving 160 patients with acute myeloid leukemia (AML) [16]. At one month after diagnosis, 28.1% exhibited clinically significant PTSD symptoms—a prevalence higher than typically observed in other cancer populations. The timing of the assessment, conducted one-month post-diagnosis, suggests that the initial diagnostic and treatment planning period may be a particularly vulnerable phase in the AML care trajectory [16,17]. Both those with and without clinically significant PTSD reported intrusion, avoidance, and hypervigilance symptoms, underscoring the need for early and routine screening in AML. In addition to the PCL-C, the study used the Brief COPE inventory to assess coping strategies and the Functional Assessment of Cancer Therapy–Leukemia (FACT-Leukemia) to measure quality of life. Higher baseline quality of life and smaller declines during chemotherapy hospitalization were associated with fewer PTSD symptoms.

Ehooman et al. conducted a multicenter prospective observational study of 269 critically ill patients with hematological malignancies admitted to intensive care units (ICUs) [17]. PTSD prevalence was 8% as measured by the IES-R. The relatively low rate may be partly attributable to the routine use of ICU diaries [14,30], which aim to aid memory reconstruction and emotional processing. Although some meta-analyses suggest only modest benefits, these findings highlight the potential value of structured, trauma-informed interventions in reducing long-term distress among ICU survivors.

Romito et al. studied 77 outpatients receiving non-deferrable chemotherapy or immunotherapy for lymphoproliferative neoplasms during the COVID-19 pandemic in Apulia, Italy—an area with intermediate incidence [18]. PTSD prevalence was 36%, the highest reported among reviewed studies, based on IES-R screening [18]. Elevated rates were plausibly linked to pandemic-related stressors such as treatment disruptions, social isolation, and heightened infection risk. Limitations include the absence of pre-pandemic baseline data and the single-institution design.

Camille et al. conducted a prospective longitudinal study of 129 patients with lymphoma, assessing PTSD symptoms with the French-validated PCL-S [19]. None met full PTSD criteria, but 23% reported subthreshold symptoms. Qualitative analysis revealed that 13.4% identified receiving the diagnosis as the primary trauma, 8% cited disclosing the illness to family, and 1.6% associated distress with treatment side effects [19]. These findings highlight the interpersonal dimensions of trauma in cancer care. Interpretation is limited by the small sample size.

Collectively, the prevalence of full-threshold PTSD among patients with hematological malignancies ranged from 8% to 36%, while subthreshold PTSD and adjustment disorders were common, often doubling or tripling the total proportion affected compared to full PTSD alone. Psychological distress frequently emerged early in the illness trajectory and was shaped by medical, interpersonal, and contextual factors. Younger age, comorbidities, active disease, and external stressors such as the COVID-19 pandemic amplified vulnerability, whereas optimism, adaptive coping, and supportive interventions (e.g., ICU diaries) appeared to mitigate trauma responses. Overall, PTSD-range symptoms are common in this population, but precise prevalence estimates remain uncertain due to methodological variability.

### 4.3. Comparative Perspective with Solid-Tumor Research

A 34-study meta-analysis in breast cancer placed overall PTSD prevalence at 9.6% (95% CI 7.9–11.5) and showed that only assessments using the most sensitive CAPS interview reached the upper teens [31]. Head-and-neck cancer, a solid tumor group with comparably intensive, disfiguring treatment, yields an intermediate figure of 17.7% for post-traumatic stress symptoms [32] while pandemic-era cohorts demonstrate context-driven spikes (e.g., 23% PTSD symptoms in French breast-cancer patients during the first COVID-19 lockdown) [33]. Thus, hematology populations appear to sit at the higher end of the cancer PTSD spectrum. Measurement heterogeneity is a shared obstacle. Both studies rely heavily on single-center, cross-sectional designs but randomized intervention trials are even more scarce in hematology research. Aligning future studies on uniform DSM-5 instruments, common time points, and multi-center recruitment would enable direct, high-confidence comparisons across cancer types.

### 4.4. Special Populations—PTSD in HSCT

HSCT is widely acknowledged as one of the most psychologically and physically demanding interventions in modern cancer care. The procedure entails high-dose chemotherapy or total body irradiation, profound immunosuppression, extended hospitalization, and the potential for life-threatening complications such as graft-versus-host disease (GVDH). These stressors occur within an atmosphere of intense emotional strain and prolonged social isolation, positioning HSCT as a high-risk setting for trauma exposure and a well-recognized context for the development of PTSD [34].

Fenech et al. conducted a secondary analysis of longitudinal data from 250 patients who underwent autologous or allogeneic HSCT while enrolled in one of two supportive care studies [21]. At six months post-transplant, 18.9% reported clinically significant PTSD symptoms, with hypervigilance, intrusion, and avoidance most frequently endorsed. Even those not meeting full diagnostic criteria often reported elevated hypervigilance and avoidance. Lower baseline quality of life (QOL), higher pre-transplant depression and anxiety, and increased anxiety during hospitalization predicted greater PTSD symptoms at follow-up. Younger age and being single were also associated with higher risk. Additional contributors may include aspects of the pre-transplant illness experience, the early post-transplant recovery phase, and complications such as GVHD [21].

El-Jawahri et al. conducted a prospective observational study of 90 HSCT recipients, with full follow-up data for 67 participants [22]. Using the PCL-C to assess PTSD symptoms six months post-transplant, the prevalence was 28.4%—substantially higher than in comparable oncology cohorts. Declines in QOL and increased depression during hospitalization were strong predictors of poorer QOL and elevated PTSD symptoms at follow-up [22]. These findings challenge the assumption that symptom burden during HSCT is short-lived, suggesting that hospitalization itself may be a traumatic event. Interventions targeting pain, fatigue, insomnia, and depression during admission may help mitigate long-term psychological consequences.

Across multiple studies—including those conducted by Fennech et al. and Springer et al.—a consistent set of predictors has emerged for post-HSCT PTSD [15,21]. Pre-transplant psychological distress, such as depression and anxiety, inadequate social support, impairments in physical functioning or reduced physical activity in the aftermath of transplantation, and negative cognitive appraisal of the transplant experience—such as interpreting it as a near-death event—have been identified as predictors of post-HSCT PTSD [21,22]. Impairments in physical functioning or reduced physical activity may elevate PTSD risk by contributing to loss of autonomy and a diminished capacity to engage in restorative behaviors [16]. Demographic factors including younger age, female gender, and lower socioeconomic status have also been linked to higher risk, although findings are less consistent.

The psychological burden extends beyond patients to caregivers, who frequently experience emotional exhaustion, burnout, and secondary trauma [16]. The demands of providing near-constant care—while managing uncertainty about relapse, complications, and prolonged dependency—can be profound.

Despite the profound distress documented among patients and caregivers, structured psychosocial support remains underutilized. Many transplant centers lack standardized PTSD screening protocols or embedded mental health services [35]. Routine psychological screening is recommended at key time points, particularly before transplantation and again at six months post-transplant, when PTSD prevalence is known to peak. Early identification enables targeted intervention, especially for patients with pre-existing psychological vulnerabilities or low baseline quality of life. A multidisciplinary approach—integrating psycho-oncologists, clinical psychologists, and social workers into transplant teams—ensures that patients receive comprehensive psychosocial support throughout treatment and survivorship.

### 4.5. Treatment Approaches and Interventions

Management of PTSD in patients with hematological malignancies is guided by an emerging but limited body of evidence, much of which is derived from broader oncological research and general PTSD treatment approaches.

#### 4.5.1. Psychological Interventions

Cognitive Behavioral Therapy (CBT)—including both exposure-based approaches and telephone-delivered formats, remains the most well-supported intervention for reducing PTSD symptoms and overall psychological distress in cancer survivors, including individuals following hematopoietic stem cell transplantation (HSCT). DuHamel and colleagues conducted a study comparing telephone-administered Cognitive Behavioral Therapy (CBT) to an assessment-only control condition in patients experiencing PTSD symptoms related to hematopoietic stem cell transplantation for hematologic or lymphoid malignancies [36]. The therapy focused on helping patients understand illness-related trauma, challenge maladaptive beliefs, gradually confront distressing memories, and develop coping strategies such as relaxation and communication skills. The majority of participants completed the intervention and reported improvements in key PTSD domains, particularly intrusive thoughts and avoidance behaviors. These benefits were sustained over time, suggesting that CBT can offer lasting support for psychological recovery following intensive cancer treatment.

Eye movement desensitization and reprocessing (EMDR) is a structured psychotherapy approach designed to help individuals process and reduce the emotional impact of traumatic memories [37]. It involves recalling distressing experiences while simultaneously engaging in bilateral stimulation, such as guided eye movements, which is thought to facilitate adaptive information processing and emotional resolution [37]. Capezzani et al. [38] conducted a study that included adult cancer patients diagnosed with PTSD, anxiety, or depression. The population consisted specifically of individuals who had experienced cancer as a traumatic event and were exhibiting clinically significant psychological symptoms. The study enrolled patients with breast cancer, colon cancer, and lymphoma, and compared the effectiveness of EMDR with cognitive behavioral therapy. This study focused on cancer-related psychological distress rather than on a specific cancer type.

Coping Strategies—Amonoo et al. found that approach-oriented coping, an active strategy involving problem-solving and cognitive-behavioral efforts to manage illness-related stress, was linked to better psychological outcomes in patients with AML [39]. Such adaptive coping has been associated with improved quality of life across cancer populations. Since coping behaviors are modifiable, these findings highlight an opportunity for behavioral interventions aimed at strengthening adaptive coping early in the illness course.

Hypnosis—Carlson et al. evaluated hypnosis for managing adverse effects such as nausea, vomiting, pain, anxiety, distress, and hot flashes. In comparison with yoga and CBT, self-hypnosis was particularly effective in lowering stress and improving quality of life. Breast cancer patients receiving preoperative hypnosis reported significantly less procedure-related anxiety and distress [40].

Mindfulness-Based Stress Reduction (MBSR)—Evidence for MBSR in hematologic malignancies is limited compared to solid tumors. A 2016 Cochrane review identified only one small randomized controlled trial in acute leukemia, showing potential benefits for quality of life and depression but with very-low-quality evidence due to methodological limitations [41]. A small prospective study in hospitalized hematology patients and caregivers found benefits in relaxation, sleep, and interest in mindfulness practice, but lacked randomization and had a limited sample size [42]. While preliminary findings suggest potential for MBSR and related practices, well-designed, hematology-specific randomized trials are needed to establish efficacy.

Trauma-Informed Care (TIC)—A growing body of literature underscores the relevance of trauma-informed care (TIC) in oncology; however, its definition, application, and evaluation remain inconsistent across cancer settings. Approaches to trauma screening vary widely, ranging from universal to targeted, with some advocating implementation regardless of trauma disclosure. Reported interventions include community-based mind–body programs, psychoeducation for at-risk adults, and family-centered care in pediatric oncology. While TIC is typically delivered by interdisciplinary teams—such as oncologists, nurses, social workers, and other providers—many studies lack detailed implementation guidance and robust feasibility evaluations [43].

TIC is guided by core principles aimed at creating a safe, respectful, and empowering care environment. These include understanding the prevalence and impact of trauma, recognizing its signs and symptoms, integrating trauma knowledge into care systems, and actively resisting re-traumatization. In hematologic oncology, these principles translate into practical strategies such as routine distress screening, transparent communication, shared decision-making, and individualized psychosocial support—particularly for high-risk patients undergoing intensive treatments. The American College of Surgeons also emphasizes patient and provider safety, trust-building, cultural sensitivity, and multidisciplinary collaboration [43].

Although some studies report improvements in patient trust, psychological well-being, and quality of life following TIC implementation, empirical evidence remains limited. Only five studies to date have documented the integration of TIC into cancer care, and none have evaluated feasibility or sustainability. Key gaps include the lack of oncology-specific conceptual models, inconsistent operationalization, limited empirical evaluation, and differing opinions on the need for formal TIC training [43].

To advance the field, future research should focus on developing and validating cancer-specific TIC frameworks, assessing their feasibility and effectiveness through rigorous study designs, and creating practical tools for clinical implementation. Incorporating both patient and provider perspectives—including preferences regarding trauma disclosure, care delivery, and communication—will be essential for meaningful and sustainable integration of TIC across the cancer care continuum.

In the context of integrated cancer care, psycho-oncology services and survivorship clinics provide added value for patients with hematologic malignancies by facilitating continuous psychological screening, early intervention, and longitudinal support across the treatment continuum [44,45]. Complementary approaches such as mind–body and complementary interventions, including mindfulness-based stress reduction, yoga, and guided relaxation techniques, offer patient-accepted strategies to manage anxiety, emotional dysregulation, and trauma-related distress. Integrating these approaches alongside evidence-based psychosocial care underscores the importance of a comprehensive, holistic model in hematologic oncology [46,47].

Evidence supports CBT as the most established intervention for PTSD in hematologic oncology, with emerging but less robust evidence for EMDR, hypnosis, and mindfulness-based approaches. Coping skills training shows promise as a modifiable resilience factor. TIC offers a promising systems-level framework but requires substantial research and operational development. Embedding these interventions within multidisciplinary hematologic cancer care has the potential to improve psychological safety, reduce trauma-related morbidity, and enhance quality of life.

#### 4.5.2. Integrative Palliative Care

A notable intervention model is the early integration of palliative care alongside standard oncology treatment, particularly during intensive phases such as induction chemotherapy or HSCT [41]. Randomized controlled trials have shown that patients receiving twice-weekly inpatient palliative care consultations during hospitalization experienced significant improvements in quality of life, along with reductions in anxiety, depression, and PTSD symptoms [48]. These patients were also more likely to engage in advance care planning discussions, reflecting improved psychological coping and enhanced communication with healthcare providers.

Early palliative care integration during intensive treatment for AML or HSCT has been associated with clinically meaningful and sustained improvements in psychological outcomes, extending up to six months post-treatment. These benefits are thought to result from more effective symptom management and the promotion of adaptive coping during periods of heightened distress. The evidence supports incorporating palliative care into the routine management of patients with high-risk hematologic malignancies, particularly those undergoing intense or prolonged hospitalization.

Collectively, these treatment modalities highlight the importance of holistic, multidisciplinary care in addressing PTSD among individuals with hematologic cancers, with an emphasis not only on symptom reduction but also on fostering psychological resilience and overall well-being throughout the cancer trajectory.

#### 4.5.3. Pharmacological Treatments

In addition to psychological interventions, pharmacologic treatments can serve as valuable adjuncts for patients with moderate to severe PTSD symptoms, particularly when distress interferes with daily functioning. Current pharmacologic management is guided by general PTSD treatment guidelines, as no studies have specifically evaluated pharmacologic strategies in patients with hematologic malignancies. Selective serotonin reuptake inhibitors (SSRIs), such as sertraline, and the serotonin–norepinephrine reuptake inhibitor (SNRIs) venlafaxine are considered first-line treatments for core PTSD symptoms [49,50]. Prazosin is often prescribed to target sleep disturbances and trauma-related nightmares [51], with alternatives including doxazosin [52] and clonidine [53]. For persistent symptoms or partial response, a second SSRI or venlafaxine may be tried. In select cases, second-generation antipsychotics may be used as augmentation—particularly if psychotic features are present—although evidence is limited and adverse effects may be significant. All pharmacologic interventions should be carefully individualized, taking into account cancer-related comorbidities, polypharmacy, and potential drug–drug interactions [49,54,55].

#### 4.5.4. The Role of Social Support

Social support is a critical protective factor influencing psychological well-being and quality of life (QOL) in patients with hematologic malignancies. Both the perceived availability and emotional quality of support networks, especially from family and friends are associated with lower psychological distress and improved psychological outcomes [56,57]. Large-scale quantitative syntheses demonstrate that perceived social support is more strongly linked to psychological adjustment than the mere receipt of support, with consistent effects across mental health outcomes such as depression, anxiety, and stress [58]. Emotional support from family and friends, as well as satisfaction with this support, is more protective for psychological distress than the frequency or quantity of contact alone [59]. Peer support, including support groups, also plays a critical role in reducing distress and enhancing self-care and health perceptions, particularly in patients who may lack strong family or partner support [60].

## 5. Limitations

This narrative has several limitations, both in its methodology and in the available evidence base, which should be considered when interpreting the findings.

This review was not registered in a protocol registry, which is acceptable for narrative reviews, but limits methodological transparency. Although a comprehensive search strategy was used across five major databases, only eight studies met the inclusion criteria, reflecting the limited and emerging nature of empirical research on PTSD in adult hematologic oncology populations. Consistent with the narrative review, no formal meta-analysis or quantitative synthesis was conducted; no standardized risk of bias assessment was conducted.

Significant gaps in the literature further constrain the conclusions that can be drawn. A major concern is the consistent underrepresentation of racial and ethnic minority populations, which limits the generalizability of findings across diverse sociocultural settings. Most studies were conducted in Western, predominantly White cohorts, with little exploration of how cultural beliefs, language barriers, or systemic inequities may shape trauma responses or access to care in marginalized groups.

Another gap is the lack of longitudinal research tracking the development and trajectory of PTSD symptoms across the cancer continuum, from diagnosis through treatment, remission, or relapse. Most existing studies are cross-sectional, which precludes insight into the evolution and persistence of trauma-related distress over time. Additionally, there is marked heterogeneity in PTSD assessment methods, with different studies employing varied screening tools (e.g., PCL-C, IES-R, CAPS) and thresholds, complicating comparisons and potentially introducing variability in reported prevalence rates.

Additionally, the exclusion of the gray literature and qualitative studies may have further narrowed the scope of this review. Given the limited and emerging nature of research on PTSD in hematological malignancies, incorporating such sources might have provided complementary insights, particularly into patient experiences and contextual factors not captured in quantitative studies.

Taken together, these limitations underscore the need for more diverse, longitudinal, and methodologically standardized research to guide trauma-informed care in hematologic oncology. Addressing these gaps will require studies with representative populations, the adoption of uniform diagnostic criteria and assessment tools, and greater clarity in distinguishing PTSD from other distress syndromes common in oncology.

## 6. Future Directions and Clinical Implications

A primary clinical recommendation is the routine screening for PTSD symptoms at multiple time points across the treatment trajectory. Early identification of at-risk individuals allows for timely mental health referrals, mitigating the risk of symptom progression and chronic impairment.

Beyond screening, the integration of trauma-informed care principles into hematology and oncology settings represents an essential paradigm shift. This approach requires clinicians to recognize trauma’s pervasive impact on behavior and health, foster emotionally safe environments, and adopt practices that are sensitive to trauma history without retraumatizing patients. Embedding mental health professionals within cancer care teams, particularly psycho-oncologists, social workers, and palliative care specialists, can facilitate these goals and help normalize psychological support as part of standard cancer care.

Digital interventions, such as app-based cognitive behavioral therapies and tele-mental health platforms, hold promises for expanding access to care, especially for rural or underserved populations. Lastly, greater focus must be placed on the psychological burden experienced by family members and caregivers, who often face their own trauma while navigating the complexities of supporting a loved one through aggressive treatments or transplant recovery. Their emotional health not only influences patient outcomes but also constitutes an unmet clinical need in its own right.

Collectively, these future directions underscore the importance of transitioning from reactive to preventive, personalized, and system-integrated models of psychosocial care in hematologic oncology. By addressing the gaps in early detection, trauma-sensitive practice, and caregiver inclusion, clinical systems can better support both survival and emotional recovery.

## 7. Conclusions

The evidence reviewed underscores the significant prevalence and impact of PTSD in individuals with hematological malignancies and those undergoing HSCT, respectively. Across multiple studies, PTSD rates ranged from approximately 15% to 35%, with many more patients experiencing subthreshold trauma symptoms or adjustment-related distress. Key risk factors include pre-transplant psychological vulnerability, impaired quality of life, lack of social support, and negative cognitive appraisal of the treatment experience. These findings reinforce that PTSD is not a marginal issue but a central psychological consequence of cancer diagnosis and treatment.

The importance of addressing PTSD in this population cannot be overstated. Psychological distress affects not only emotional well-being but also treatment adherence, quality of life, immune functioning, and long-term survivorship outcomes. As such, PTSD should be regarded as a core dimension of holistic cancer care, alongside physical, social, and spiritual domains. Incorporating routine psychological screening, trauma-informed care models, and integrated psychosocial support services is essential to meet the complex needs of this vulnerable group.

## Figures and Tables

**Table 1:** Summary of included studies—PTSD and trauma-related disorders in hematologic malignancies.

Serial Number	Study	Design	Sample (n)	Assessment—Scales Used	Timing of Assessment	PTSD Prevalence	Other Observations	**Country**
1	Prevalence and Positive Correlates of Posttraumatic Stress Disorder Symptoms among Chinese Patients with Hematological Malignancies: A Cross-Sectional Study (2015) [14]	Observational cross-sectional study	225	PTSD Checklist-Civilian Version (PCL-C)	Not reported	10.7%	Optimism was measured as a personality trait in the present study and shown to have a positive effect on PTSD symptoms.	China
2	Symptoms of posttraumatic stress disorder and adjustment disorder in hematological cancer patients with different treatment regimes (2023) [15]	Observational cross-sectional study	285	PCL-C Adjustment Disorder–New Module 20 (ADMN-20)	Note reported	26 (9.3%) met criteria for PTSD 66 (23.7%) met criteria for subthreshold PTSD 40 (14.2%) met criteria for adjustment disorder (AjD)	Factors associated with elevated symptomatology were younger age, physical comorbidity and active disease	Germany
3	Posttraumatic stress disorder symptoms in patients with acute myeloid leukemia (2021) [16]	Secondary analysis/supportive care trial	160	PCL-C	1 month after diagnosis	40 patients—25%	The checklist was used to assess PTSD symptoms at 1 month after AML diagnosis. The Brief COPE and the Functional Assessment of Cancer Therapy-Leukemia were to assess coping and quality of life (QOL), respectively.	United States of America (USA)
4	Long-term health-related quality of life of critically ill patients with haematological malignancies: a prospective observational multicenter study (2019) [17]	Cross-sectional study	269	Impact of Event Scale (IES-R)	Not reported	22 (8%) patients had an IES score greater than 35 points, which is considered the threshold for PTSD.	The use of an ICU diary in many participating centers may have participated in the low incidence of PTSD as an ICU diary has been associated with a significant reduction in PTSD symptoms in critical illness survivors	France, Belgium
5	Psychological Distress in Outpatients with Lymphoma During the COVID-19 Pandemic (2020) [18]	Prospective observational study	77	IES-R	Not reported	36% (n. 28)	Study conducted during COVID-19 pandemic	Italy
6	Posttraumatic Stress Disorder Symptoms in Lymphoma Patients: A Prospective Study (2020) [19]	Prospective observational study	129	PCL-C	Not reported	0 patients met full PTSD criteria 29 (23%) subthreshold PTSD	13.4% related it to receiving the diagnosis of lymphoma, 8% to telling family members, and 1.6% to adverse effects.	France

**Table 2:** Summary of included studies—PTSD after HSCT.

Serial Number	Study	Design	Sample (n)	Assessment—Scales Used	PTSD Prevalence	Timing of Assessment	Country
1	Post-Traumatic Stress Symptoms in Hematopoietic Stem Cell Transplant Recipients (2021) [21]	Secondary analysis of randomized controlled trial	206	PCL-C	18.9% (39)	6 months after transplantation	USA
2	Quality of life and mood predict posttraumatic stress disorder after hematopoietic stem cell transplantation (2016) [22]	Prospective observational study	90	PCL-C	At 6 months, 28.4% of participants met the criteria for PTSD	Data at 6 months were available for 67 participants.	USA

## Data Availability

The contributions presented in this study are included in the article. Further inquiries can be directed to the corresponding author.
